# Supplementary material for: The BCKDK inhibitor BT2 is a chemical uncoupler that lowers mitochondrial ROS production and de novo lipogenesis
Source: J Biol Chem. 2024 Jan 30;300(3):105702. doi: 10.1016/j.jbc.2024.105702 (PMC10910128; doi:10.1016/j.jbc.2024.105702)
Supplement: Supporting information [file mmc1.pdf]

SUPPORTING INFORMATION

"The BCKDK inhibitor BT2 is a chemical uncoupler that lowers mitochondrial ROS production and *de novo* lipogenesis"

Includes:

Supporting Information Figure S1 – BT2 uncouples in cultured cells with varying levels of BCAA oxidation.

Supporting Information Figure S2 – 200μM BT2 results in proton conductance measurements that cannot be accurately quantified

Supporting Table 1 – ISA modeled values and 95% confidence intervals for individual technical replicates

Supporting Information Figure S1 – BT2 uncouples in cultured cells with varying levels of BCAA oxidation.

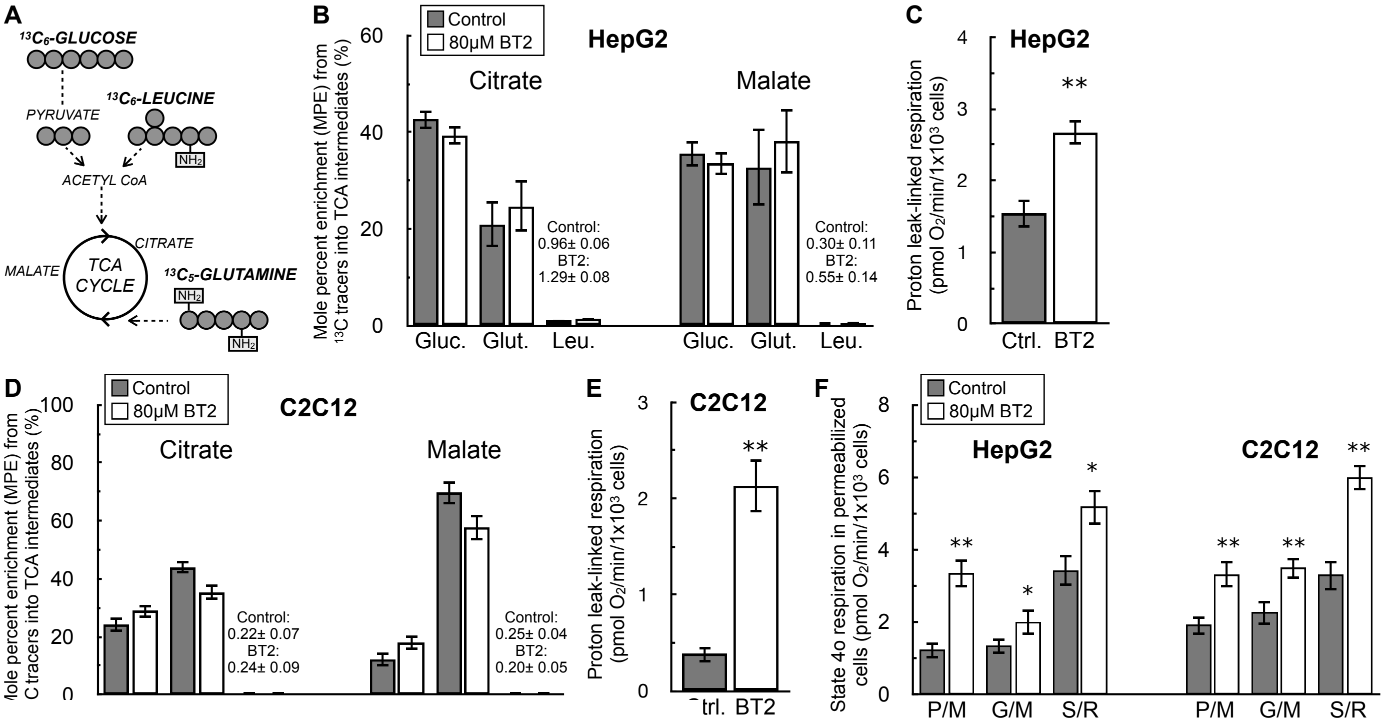

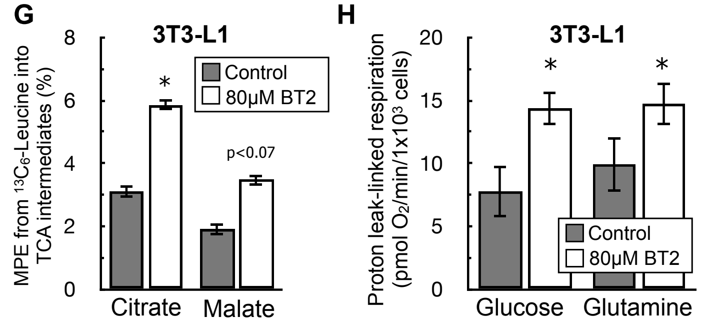

Supporting Information Figure S1 – BT2 uncouples in cultured cells with varying levels of BCAA oxidation. (A)-(E): BT2 uncoupled in cells with little-to-no measurable BCKDK activity. **(A)** Schematic showing diagram for measuring relative incorporation of glucose, leucine, and glutamine using ^{13}C-labeled tracers. **(B)** Mole percent enrichment from labeled glucose, glutamine, and leucine into the TCA cycle intermediates malate and citrate for HepG2 cells in vehicle controls (filled bars) or cells treated with 80μM BT2 (open bars). (n=3 biological replicates). **(C)** Uncoupled respiration in HepG2 cells offered glucose, glutamine, and pyruvate in DMEM assay medium. (n=4 biological replicates). **(D)** Mole percent enrichment from labeled glucose, glutamine, and leucine into the TCA cycle intermediates malate and citrate for C2C12 myoblasts in vehicle controls (filled bars) or treated with 80μM BT2 (open bars). (n=4 biological replicates). **(E)** Uncoupled respiration in C2C12 cells offered glucose, glutamine, and pyruvate in DMEM assay medium. (n=3 biological replicates). **(F)** BT2 uncouples respiration in permeabilized cells with substrates that bypass BCKDK: State 4_o respiration in permeabilized HepG2 (left) and C2C12 (right) cells in vehicle controls (filled bars) or cells treated with 80μM BT2 (open bars). Permeabilized cells were offered pyruvate/malate (P/M), glutamate/malate (G/M), or succinate/rotenone (S/R). [n=4 (C2C12) or n=5 (HepG2) biological replicates]. **(G)-(H):** As a 'positive control' for measurable leucine oxidation, 3T3-L1 adipocytes show BT2 behaves as expected, but uncouples irrespective of the respiratory substrate provided. **(G)** Mole percent enrichment from uniformly labeled $^{13}C_6$-leucine in differentiated 3T3-L1 adipocytes in vehicle controls (filled bars) or cells treated with 80μM BT2 (open bars). (n=4 biological replicates) **(H)** Uncoupled respiration in differentiated 3T3-L1 adipocytes in the presence or absence of BT2 as before. Cells were assayed in a simple-salts, Krebs-Henseleit buffer supplemented with either glucose or glutamine. (n=3 biological replicates). All data are presented as mean ± S.E.M.

Supporting Information Figure S2 – 200μM BT2 results in proton conductance measurements that cannot be accurately quantified

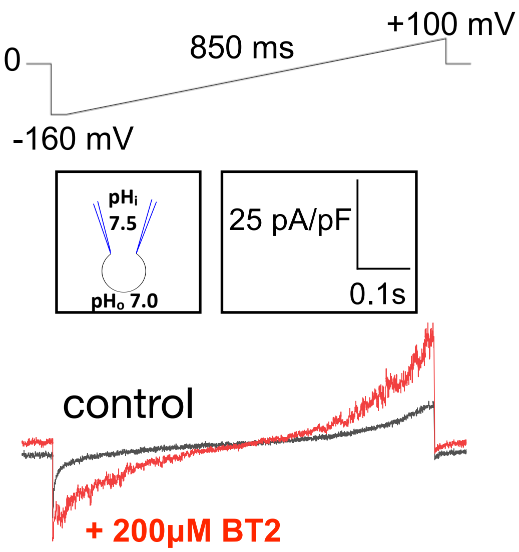

Supporting Information Figure S2: Patch-clamp electrophysioloigy with cardiac mitoplasts demonstrates that 200μM BT2 yields a bigger current amplitude than 100μM, but disrupts the integrity of the inner membrane. As such, a proton conductance cannot be reliably quantified.

Supporting Information Table S1: ISA modeled values and 95% confidence intervals for individual technical replicates.

Sample	Measurement	Value	Lower range	Upper range		Sample	Measurement	Value	Lower range	Upper range
N1-NT1	D(M2-AcCoA)	0.309	0.264	0.353		N3-NT1	D(M2-AcCoA)	0.558	0.509	0.605
	D(M1-AcCoA)	0.027	0.006	0.052			D(M1-AcCoA)	0.034	0.010	0.066
	1-D(AcCoA)	0.665	0.621	0.708			1-D(AcCoA)	0.408	0.360	0.457
	g(t) palmitate	0.419	0.375	0.459			g(t) palmitate	0.456	0.408	0.498
	TOTAL AcCoA contribution	0.335	0.270	0.405			TOTAL AcCoA contribution	0.592	0.519	0.671
N1-NT2	D(M2-AcCoA)	0.288	0.240	0.335		N3-NT2	D(M2-AcCoA)	0.292	0.236	0.348
	D(M1-AcCoA)	0.027	0.006	0.055			D(M1-AcCoA)	0.030	0.005	0.065
	1-D(AcCoA)	0.685	0.638	0.731			1-D(AcCoA)	0.677	0.624	0.730
	g(t) palmitate	0.394	0.349	0.435			g(t) palmitate	0.354	0.307	0.396
	TOTAL AcCoA contribution	0.315	0.246	0.390			TOTAL AcCoA contribution	0.323	0.241	0.412
N1-NT3	D(M2-AcCoA)	0.244	0.194	0.291		N3-NT3	D(M2-AcCoA)	0.326	0.248	0.398
	D(M1-AcCoA)	0.032	0.009	0.064			D(M1-AcCoA)	0.030	0.000	0.078
	1-D(AcCoA)	0.724	0.678	0.769			1-D(AcCoA)	0.644	0.574	0.715
	g(t) palmitate	0.395	0.348	0.437			g(t) palmitate	0.262	0.211	0.306
	TOTAL AcCoA contribution	0.276	0.204	0.355			TOTAL AcCoA contribution	0.356	0.248	0.476
N1-BT2-1	D(M2-AcCoA)	0.322	0.255	0.385		N3-BT2-1	D(M2-AcCoA)	0.327	0.282	0.372
	D(M1-AcCoA)	0.029	0.000	0.070			D(M1-AcCoA)	0.023	0.002	0.048
	1-D(AcCoA)	0.650	0.588	0.712			1-D(AcCoA)	0.650	0.605	0.694
	g(t) palmitate	0.285	0.236	0.329			g(t) palmitate	0.366	0.325	0.403
	TOTAL AcCoA contribution	0.350	0.255	0.455			TOTAL AcCoA contribution	0.350	0.284	0.420
N1-BT2-2	D(M2-AcCoA)	0.299	0.221	0.370		N3-BT2-2	D(M2-AcCoA)	0.295	0.223	0.362
	D(M1-AcCoA)	0.030	0.000	0.079			D(M1-AcCoA)	0.030	0.000	0.075
	1-D(AcCoA)	0.671	0.603	0.739			1-D(AcCoA)	0.675	0.611	0.739
	g(t) palmitate	0.258	0.209	0.303			g(t) palmitate	0.235	0.191	0.274
	TOTAL AcCoA contribution	0.329	0.221	0.449			TOTAL AcCoA contribution	0.325	0.223	0.437
N1-BT2-3	D(M2-AcCoA)	0.231	0.076	0.320		N3-BT2-3	D(M2-AcCoA)	0.316	0.245	0.383
	D(M1-AcCoA)	0.046	0.004	0.169			D(M1-AcCoA)	0.028	0.000	0.072
	1-D(AcCoA)	0.723	0.646	0.826			1-D(AcCoA)	0.655	0.591	0.720
	g(t) palmitate	0.275	0.218	0.325			g(t) palmitate	0.248	0.202	0.289
	TOTAL AcCoA contribution	0.277	0.079	0.489			TOTAL AcCoA contribution	0.345	0.245	0.455
N1-DNP-1	D(M2-AcCoA)	0.355	0.236	0.455		N3-DNP-1	D(M2-AcCoA)	0.551	0.483	0.615
	D(M1-AcCoA)	0.024	0.000	0.095			D(M1-AcCoA)	0.023	0.000	0.062
	1-D(AcCoA)	0.621	0.524	0.721			1-D(AcCoA)	0.426	0.360	0.495
	g(t) palmitate	0.197	0.141	0.247			g(t) palmitate	0.111	0.088	0.134
	TOTAL AcCoA contribution	0.379	0.236	0.550			TOTAL AcCoA contribution	0.574	0.483	0.677
N1-DNP-2	D(M2-AcCoA)	0.387	0.243	0.505		N3-DNP-2	D(M2-AcCoA)	0.338	0.000	0.483
	D(M1-AcCoA)	0.022	0.000	0.105			D(M1-AcCoA)	0.031	0.000	0.287
	1-D(AcCoA)	0.591	0.476	0.713			1-D(AcCoA)	0.631	0.492	0.993
	g(t) palmitate	0.171	0.114	0.221			g(t) palmitate	0.175	0.110	0.231
	TOTAL AcCoA contribution	0.409	0.243	0.610			TOTAL AcCoA contribution	0.369	0.000	0.770
N1-DNP-3	D(M2-AcCoA)	0.346	0.201	0.456		N3-DNP-3	D(M2-AcCoA)	0.295	0.178	0.386
	D(M1-AcCoA)	0.025	0.000	0.109			D(M1-AcCoA)	0.029	0.000	0.100
	1-D(AcCoA)	0.629	0.524	0.743			1-D(AcCoA)	0.677	0.592	0.766
	g(t) palmitate	0.178	0.123	0.228			g(t) palmitate	0.172	0.127	0.212
	TOTAL AcCoA contribution	0.371	0.201	0.565			TOTAL AcCoA contribution	0.323	0.178	0.486
N2-NT1	D(M2-AcCoA)	0.224	0.187	0.260		N4-NT1	D(M2-AcCoA)	0.346	0.306	0.386
	D(M1-AcCoA)	0.031	0.012	0.054			D(M1-AcCoA)	0.023	0.004	0.045
	1-D(AcCoA)	0.746	0.711	0.780			1-D(AcCoA)	0.631	0.591	0.672
	g(t) palmitate	0.395	0.358	0.430			g(t) palmitate	0.378	0.340	0.412
	TOTAL AcCoA contribution	0.254	0.198	0.314			TOTAL AcCoA contribution	0.369	0.309	0.431
N2-NT2	D(M2-AcCoA)	0.387	0.337	0.436		N4-NT2	D(M2-AcCoA)	0.523	0.475	0.571
	D(M1-AcCoA)	0.024	0.002	0.052			D(M1-AcCoA)	0.029	0.007	0.059
	1-D(AcCoA)	0.589	0.540	0.638			1-D(AcCoA)	0.447	0.399	0.496
	g(t) palmitate	0.420	0.373	0.462			g(t) palmitate	0.429	0.383	0.470
	TOTAL AcCoA contribution	0.411	0.339	0.488			TOTAL AcCoA contribution	0.553	0.481	0.630
N2-NT3	D(M2-AcCoA)	0.249	0.203	0.293		N4-NT3	D(M2-AcCoA)	0.435	0.381	0.489
	D(M1-AcCoA)	0.030	0.008	0.059			D(M1-AcCoA)	0.023	0.000	0.052
	1-D(AcCoA)	0.721	0.679	0.763			1-D(AcCoA)	0.541	0.487	0.595
	g(t) palmitate	0.393	0.350	0.432			g(t) palmitate	0.412	0.363	0.455
	TOTAL AcCoA contribution	0.279	0.212	0.352			TOTAL AcCoA contribution	0.459	0.381	0.541
N2-BT2-1	D(M2-AcCoA)	0.398	0.295	0.492		N4-BT2-1	D(M2-AcCoA)	0.300	0.246	0.352
	D(M1-AcCoA)	0.029	0.000	0.094			D(M1-AcCoA)	0.026	0.001	0.058
	1-D(AcCoA)	0.573	0.480	0.669			1-D(AcCoA)	0.674	0.624	0.724
	g(t) palmitate	0.231	0.172	0.281			g(t) palmitate	0.266	0.226	0.302
	TOTAL AcCoA contribution	0.427	0.295	0.586			TOTAL AcCoA contribution	0.326	0.247	0.411
N2-BT2-2	D(M2-AcCoA)	0.282	0.203	0.354		N4-BT2-2	D(M2-AcCoA)	0.317	0.266	0.367
	D(M1-AcCoA)	0.032	0.000	0.084			D(M1-AcCoA)	0.023	0.000	0.053
	1-D(AcCoA)	0.685	0.619	0.753			1-D(AcCoA)	0.660	0.611	0.709
	g(t) palmitate	0.279	0.227	0.325			g(t) palmitate	0.232	0.198	0.263
	TOTAL AcCoA contribution	0.315	0.203	0.437			TOTAL AcCoA contribution	0.340	0.266	0.420
N2-BT2-3	D(M2-AcCoA)	0.369	0.271	0.459		N4-DNP-1	D(M2-AcCoA)	0.365	0.247	0.468
	D(M1-AcCoA)	0.030	0.000	0.091			D(M1-AcCoA)	0.026	0.000	0.093
	1-D(AcCoA)	0.601	0.514	0.690			1-D(AcCoA)	0.609	0.508	0.714
	g(t) palmitate	0.252	0.193	0.303			g(t) palmitate	0.183	0.133	0.229
	TOTAL AcCoA contribution	0.399	0.271	0.549			TOTAL AcCoA contribution	0.391	0.247	0.561
N2-DNP-1	D(M2-AcCoA)	0.464	0.319	0.590		N4-DNP-2	D(M2-AcCoA)	0.340	0.271	0.408
	D(M1-AcCoA)	0.021	0.000	0.117			D(M1-AcCoA)	0.019	0.000	0.056
	1-D(AcCoA)	0.515	0.390	0.650			1-D(AcCoA)	0.641	0.576	0.706
	g(t) palmitate	0.150	0.094	0.201			g(t) palmitate	0.163	0.129	0.195
	TOTAL AcCoA contribution	0.485	0.319	0.707			TOTAL AcCoA contribution	0.359	0.271	0.464
						N4-DNP-3	D(M2-AcCoA)	0.316	0.239	0.388
							D(M1-AcCoA)	0.021	0.000	0.064
							1-D(AcCoA)	0.663	0.594	0.732
							g(t) palmitate	0.162	0.126	0.194
							TOTAL AcCoA contribution	0.337	0.239	0.452
